# Identification and Comparison of Anti-Inflammatory Ingredients from Different Organs of *Lotus Nelumbo* by UPLC/Q-TOF and PCA Coupled with a NF-κB Reporter Gene Assay

**DOI:** 10.1371/journal.pone.0081971

**Published:** 2013-11-29

**Authors:** Mengge Zhou, Min Jiang, Xuhui Ying, Qingxin Cui, Yanqi Han, Yuanyuan Hou, Jie Gao, Gang Bai, Guoan Luo

**Affiliations:** 1 State Key Laboratory of Medicinal Chemical Biology and College of Pharmacy, Tianjin Key Laboratory of Molecular Drug Research, Nankai University, Tianjin, China; 2 State Key Laboratory of Medicinal Chemical Biology and College of Pharmacy, Nankai University, Tianjin, China; 3 Department of Chemistry, Tsinghua University, Beijing, China; Duke University Medical Center, United States of America

## Abstract

*Lotus nelumbo (LN*) (*Nelumbo nucifera* Gaertn.) is an aquatic crop that is widely distributed throughout Asia and India, and various parts of this plant are edible and medicinal. It is noteworthy that different organs of this plant are used in traditional herbal medicine or folk recipes to cure different diseases and to relieve their corresponding symptoms. The compounds that are contained in each organ, which are named based on their chemical compositions, have led to their respective usages. In this work, a strategy was used to identify the difference ingredients and screen for Nuclear-factor-kappaB (NF-κB) inhibitors with anti-inflammatory ability in *LN*. Seventeen main difference ingredients were compared and identified from 64 samples of 4 different organs by ultra-performance liquid chromatography that was coupled with quadrupole/time of flight mass spectrometry (UPLC/Q-TOF-MS) with principal component analysis (PCA). A luciferase reporter assay system combined with the UPLC/Q-TOF-MS information was applied to screen biologically active substances. Ten NF-κB inhibitors from *Lotus plumule (LP*) extracts, most of which were isoquinoline alkaloids or flavone C-glycosides, were screened. Heat map results showed that eight of these compounds were abundant in the *LP*. In conclusion, the *LP extracts* were considered to have the best anti-inflammatory ability of the four *LN* organs, and the chemical material basis (CMB) of this biological activity was successfully validated by multivariate statistical analysis and biological research methods.

## Introduction

*Lotus nelumbo (LN*) (*Nelumbo nucifera* Gaertn) is an aquatic crop that is widely distributed throughout Asia and India, and various parts of this plant are edible and medicinal. Because this plant can be used as common vegetables, cooking oil, tea and healthcare products, the lotus plumule (LP), louts leaf (LL), lotus seed (LS), and lotus rhizome (LR) are favored by people for their nutritional values [1,2]. Meanwhile, the different organs of this plant are also used in traditional herbal medicine or folk recipes to cure different diseases and to relieve their corresponding symptoms. However, significant differences between the types and levels of chemicals from each of the different organs are found [3,4], which causes some variance in its medicinal purposes and biologically active capacities [5]. In other words, the chemical material basis (CMB) of *Lotus nelumbo* leads to different plant parts being used for different applications. The *LP* has been recognized as an anti-inflammatory agent in traditional Chinese medicine (TCM), and it has been especially used in the treatment of various inflammatory-related diseases such as spontaneous inflammation, high fever and myocarditis [6,7]. *LL* organs are particularly important for their antioxidation and antiobesity pharmacological properties [8,9]; while, research has indicated that the *LS* and *LR* are rich in nutrients such as proteins, amino acids, unsaturated fatty acids and minerals and are mainly used as functional and healthy foods [10,11]. Currently, no method has been established to reliably identify the key constituents of the different organs of *LN*, and no reported systematic and comprehensive literature has determined the CMB and the specific biological ability for these organs.

With the development and popularity of some technologies, especially the application of liquid-liquid extraction and solid phase extraction (SPE) [12,13], new approaches and opportunities for the study of natural plants have been provided. Because the commonly used methods for extraction and separation, i.e., HPLC, UPLC, MS, or other analytical instruments, are more efficient and consume less organic solvents, they have been developed as efficient methods for plant-characteristic-constituents analysis [14-16]. Due to the complexity of the chemical compositions of different plant samples, the traditional integration methods [17] have been unable to simultaneously take into account the difference between content and activity. Yet, there is a definite need for a new strategy that can handle a large number of data while comparing the object products of different active ingredients of a particular bioactivity from different samples [18,19]. PCA is a commonly used, multivariate statistical method that is used to distinguish chemical components by cluster analysis and factorial k-means analysis of relatively large data sets that are acquired from HPLC-DAD, UPLC-MS and spectroscopic techniques [20-22]. In this study, a tandem technology with subtle integration of UPLC-Q/TOF-MS, PCA and a NF-κB luciferase reporter gene assay were used for screening NF-κB inhibitors [23]. NF-κB is a transcription factor expressed in numerous cell types, which plays a key role in the immune and inflammatory responses. Many molecules involved in immune response and early stages of inflammation are regulated by NF-κB[24,25]. The luciferase reporter gene assay was established for screening of the anti-inflammatory active ingredients from the perspective of regulation NF-κB, adapting to the demand of large amount of data.

In this study, the difference ingredients from *LP, LL, LS* and *LR* were identified and compared by PCA combined with chemical characterization analysis by UPLC-Q/TOF-MS. Simultaneously, the NF-kB inhibitors were found through screening. From the Heat map results, it was clear that the NF-κB inhibitors varied and were the most abundant in the *LP*. This analytical strategy was successful and provided solid evidence that the *LP* was better for anti-inflammation than the other organs.

## Materials and Methods

### 1. Ethics Statement

This study was approved by the Institutional Review Board at the Nankai University. HEK 293 cells and BEAS-2B cells were obtained from the American Type Culture Collection (Rockville, MD). This field study did not involve any endangered or protected species, and all the *Lotus nelumbo* we selected were common species in China. All sixty-four samples from four different organs of *LN* were purchased from commercially available Herb Drug Company including Anguo Chang'an Chinese Medicinal Herbs Co., Ltd., Anguo Qizhou Chinese Traditional Medicine Electuary Co., Ltd., and Tianjin Zhongxin Pharmaceutical Group Co., Ltd.. The origins of the plants are involving five different agricultural regions (Hebei, Hunan, Hubei, Shandong, and Fujian provinces, China). And no specific permission was required.

### 2. Chemicals and reagents

Acetonitrile for LC and MS analysis was of LC grade and from Fisher (Pittsburgh, PA, USA); formic acid (98%) for MS analysis was LC grade and from Acros Organics (Geel, Belgium); and water for LC and MS analysis was purified by a Milli-Q academic water purification system (Millipore, Mifoed, MA, USA). Higenamine, Isoliensinine, Neferine, Nuciferine and Hirsutrin were purchased from Yifang S&T (Tianjin, China). The reporter plasmid pGL4.32 and reporter vector plasmid pRL-TK were purchased from Promega (WI, USA). Human TNF-α was obtained from PeproTech (Rocky Hill, USA). Dexamethasone was purchased from the Sigma Chemical Co. (St Louis, MO, USA). All reagents for cell culture were purchased from GibcoBRL Life Technologies (Rockville, MD, USA).

### 3. Plant Materials

Sixty-four samples from four different organs of *LN* (Samples 1 through 16 corresponded to *LP*; samples 17 through 32 corresponded to *LS*; samples 33 through 48 corresponded to *LL*; and samples 49 through 64 corresponded to *LR*) were collected, and identified by Professor Tiejun Zhang from the Tianjin Institute of Pharmaceutical Research. The dried samples were stored at -20°C, and after removal of impurities by screening, the samples were ground into powder (100-mesh).

### 4. Cell culture

HEK 293 cells, Human embryonic kidney 293 cells, were obtained from the American Type Culture Collection (Rockville, MD). HEK 293 were grown in Dulbecco’s modified Eagle’s medium (DMEM) (Gibco BRL) containing 10% fetal bovine serum (FBS) (Gibco BRL), 100 U/mL of penicillin and 0.1 mg/mL of streptomycin. BEAS-2B, a cell line that was derived from human bronchial epithelial cells, was obtained from the American Type Culture Collection (Rockville, MD). BEAS-2B cells were cultured using DMEM/F12 medium that was supplemented with 10% FBS, 100 U/mL of penicillin and 0.1 mg/mL of streptomycin. Both cell types were maintained at 37 °C in 5% CO_2_, fed every 3 days and sub-cultured once they reached 80–90% confluency.

### 5. Sample preparation for analysis

The obtained dried powders (0.2 g) were accurately weighed and added to 10 mL of 75% aqueous methanol in a conical flask. Subsequently, ultra-sonication (40 kHz) was performed at room temperature for 30 min. After extraction, the extract was centrifuged at 10285 g for 15 min. The supernatant was filtered through a 0.22-μm membrane filter before injection into the UPLC-MS system for analysis. All solutions were stored in the refrigerator at 4 °C and allowed to warm at room temperature before analysis. The filtrate (0.5 ml) was evaporated to dryness in a vacuum drying oven, dissolved in 3 mL of cell culture medium and used as the high dosage for the dual-luciferase reporter assay. The medium and low dosages were diluted three or nine times, respectively.

### 6. Analysis of difference ingredients

#### 6.1. UPLC-Q/TOF MS methods

A Waters Acquity UPLC instrument system (Waters Co., USA) that was equipped with a photo diode array detector (DAD) (190-400 nm) was used for analysis, and the system was controlled by the MassLynx V4.1 software (Waters Co., USA). Separations were performed using a Waters Acquity UPLC BEH C_18_ column (2.1 mm×100 mm, 1.7 μm) at 35 °C, and a gradient elution of acetonitrile (A) and 0.1% formic acid (B) was performed as follows: 2% A was maintained from 0 to 1.9 min, A was increased to 5% from 1.9 to 2 min and was maintained at this concentration from 2 to 6 min, A was increased to 10 % from 6 to 10 min, A was increased to 15 % from 10 to 15 min and this concentration was maintained from 15 to 17 min, A was increased to 25% from 17 to 18 min, A was increased to 30% from 18 to 20 min, A was increased to 45% from 20 to 21 min, and A was increased to 100% from 21 to 25 min. The flow rate was set at 0.4 mL/min, and the injection volume of the test sample was 2 μL. Subsequently, the UPLC fractions were partly collected in a 96-well, deep-well plate every 0.5 min and then evaporated. The residues were dissolved in 100 μL of cell culture medium for the cell bioactivity assay.

Mass spectrometry was performed on a Waters Q/TOF Premier Mass Spectrometer with an electrospray ionization system (Waters MS Technologies, Manchester, UK), and the ESI-MS spectra were acquired in the negative and positive ion modes. The conditions for ESI-MS analysis were as follows: the capillary voltage was set to 2.5 kV in the negative-mode and at 3.0 kV in the positive-ion mode. The sample cone voltage was set to 30 V, the desolvation gas flow was set to 600 L/h at a desolvation temperature of 300 °C, the cone gas was set to 50 L/h, and the source temperature was 100 °C. The Q/TOF Premier acquisition rate was 0.1 s with a 0.02 s inter-scan delay. The MS spectra were acquired from 50 to 1200 Da, and leucine-enkephalinamide acetate (LEA) was used as the lock mass (m/z 555.2931 in ESI^+^ and 553.2775 in ESI^-^) at a concentration of 200 ng/mL and a flow rate of 0.2 μL/min.

#### 6.2. PCA and Identification of the Substances

PCA was used to differentiate the characteristic components of the different organs of *LN*. UPLC Q-TOF MS data were imported into Markerlynx, and the data from Markerlynx were directly imported into the Simca-P software (version 11.5, Demo, Umetrics, Umea, Sweden) to ensure a smooth fermentation process. PCA was used to distinguish the principal components (PCs), but the identification of each substance was also operated in Markerlynx and analyzed according to the UV data, primary and secondary mass spectrometry, and in relation to the related literature.

### 7. Screening of NF-κB inhibitors by the dual-luciferase reporter assay

In each well, HEK 293 cells were co-transfected with the NF-κB luciferase reporter plasmid pGL4.32 (100 ng) (Promega WI, USA) and Renilla luciferase reporter vector pRL-TK plasmid (Promega) (9.6 ng). The transfection was performed for 24 h using Lipofectamine 2000 according to the manufacturer’s instructions, and the medium was replaced with fresh, serum-free medium 24 h before the experiments. The cells were then pretreated with drugs and stimulated by TNF-α (10 ng/mL) for 6 h. 

The separated UPLC fractions or active ingredients (Higenamine, Isoliensinine, Neferine, and Nuciferine) were appropriately diluted and dissolved in 100 μL of cell culture medium for the cell bioactivity assay [23]. After the samples were stimulated, the HEK 293 cells were washed, lysed, and assayed for luciferase activity using a dual-luciferase reporter assay system (Promega) according to the manufacturer’s instructions. The relative luciferase activity was obtained by normalizing the firefly luciferase activity against the activity of the internal Renilla luciferase control (Modulus™, Turner BioSystems, USA).

### 8. Bioactivity assay for IL-6 and IL-8

Commercial enzyme-linked immunosorbent assay (ELISA) kits (Pierce/Endogen, Rockford, IL) were used to measure the release of IL-6 and IL-8 from the supernatants of BEAS-2B cells after drug and TNF-α stimulation. The absorbance of each sample was then measured at 450 nm using a Bio-Rad Model 680 micro-plate reader, and the levels of IL-6 and IL-8 were determined using standard curves and expressed as pg/mL.

### 9. Analysis of relative content

The relative content of each compound in different organs of the plant was visualized by Heat map. Peak areas of target compounds were exported from Markerlynx followed by normalization. The normalized data were processed by Cluster 3.0 (adapted by Michiel de Hoon from Cluster, written by Michael Eisen)[26], and the result was visualized by Java Treeview 1.1.6 R2 (written by Michael Eisen).

### 10. Statistical analysis

Statistical analysis was performed using the SPSS sorftware, and data were presented as the mean ± SEM. One-way ANOVA was used to determine the significance of differences between groups of data, and this analysis was followed by a LSD/Dunnett-T3 post hoc test. Values of P < 0.05 were accepted as significant.

## Results and Discussion

### 1. PCA and Identification of difference ingredients

**Figure 1:**
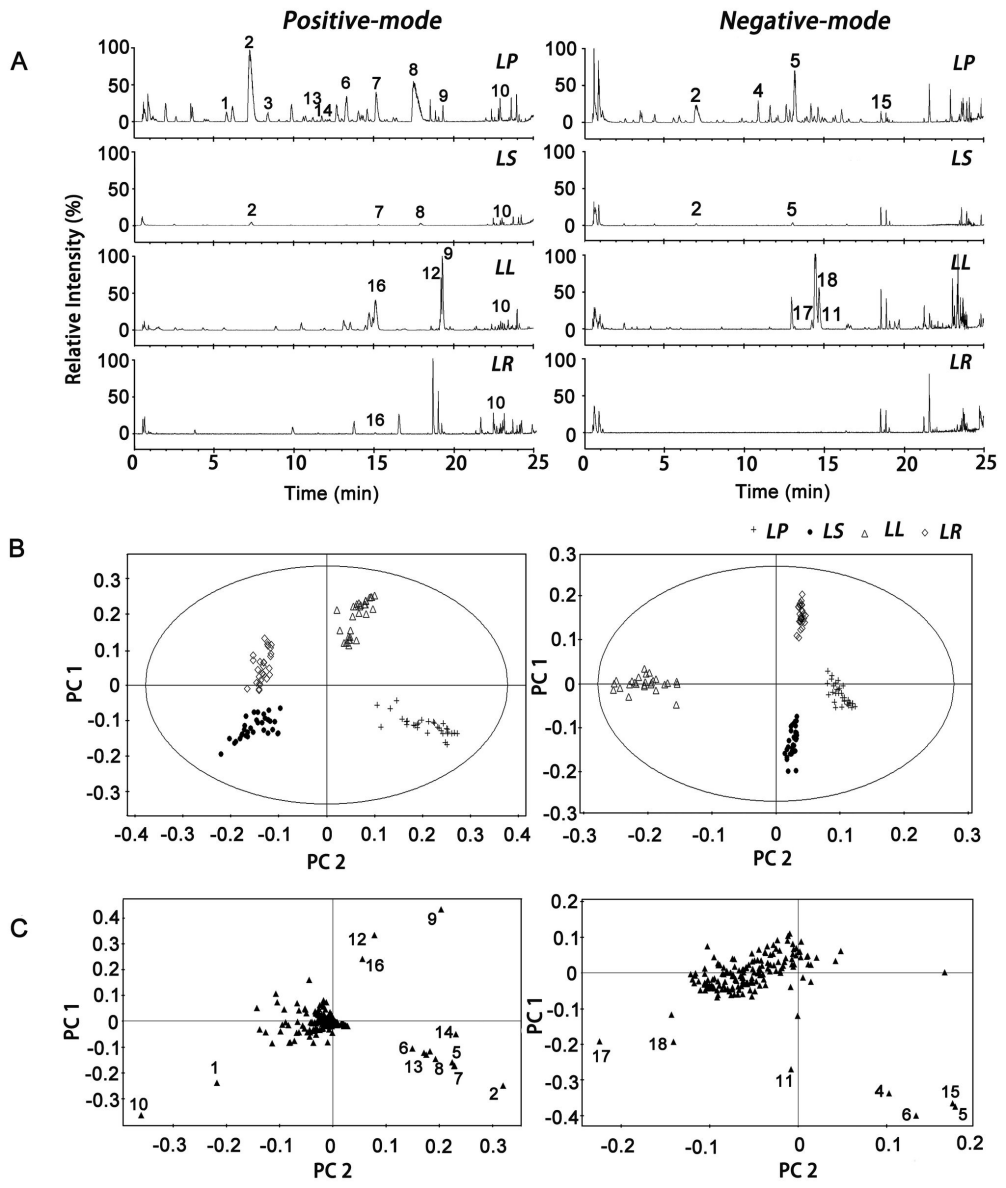
Results of PCA and the BPI chromatograms of different organs of *LN*. BPI chromatograms (**A**), score plots (**B**) and Loading plots (**C**) in the positive and negative ESI mode are shown. The peak numbers, significance values and relative content (MAX.) are consistent with those in Table 1(A and B).

After investigating the influence of powder size, ultrasonic intensity, extract time, and extraction efficiency, the intra- and inter-day precisions of the UPLC method were determined. The RSDs of the relative retention time and relative peak area were below 3%. The sample and solvent stability were also tested during this analysis period. The BPI chromatograms from the different organs under these conditions in the negative- and positive-ion modes are shown in Figure 1A. The PCA that was carried out by the MarkerLynx software was used to distinguish the products of different organs based on fingerprints, and the PCA score plots are displayed in Figure 1B. A clear discrimination between the different organs of *LN* was observed in the PCA score plots, where each coordinate was represented. All of the samples could be classified into four clusters (i.e., *LP, LS, LL* or *LR*), and no misclassification of these four groups occurred, which indicated that the samples in this organ were significantly different from those in other organs.

The contribution of each principal component is described in the loading plots (Figure 1C). The distance of the marker from the origin of the loading plots is scaled to a value that is between 0 and 1; and a higher value indicates a more significant marker. It is clearly visible that **17** compounds contributed strongly to the clusters and were used as the difference ingredients. This result also provided the maximum content of each difference ingredient in the four organs and is displayed in Table 1. As shown in Figure 1(A), the base-peak intensity (BPI) chromatograms were significantly different for the four organs of *LN.*

**Table 1:** MS/MS data from ESI-MS and identification of the PCA results and the bioactive compounds of *LP* in positive mode (A) and MS/MS data from ESI-MS and identification of the PCA results and the bioactive compounds of *LP* in negative mode (B).

**A**	**Peak NO.**	**Significance**	**t_R_(min)**	**Identification**	**MS [M+H]^*+*^**		**HPLC-ESI-*MSn* m/z**	**Composition**		**Substance Class**		**MAX.**
	1	0.3687	6.00	Higenamine **^*a*^**	272.1256		272[M+H]**^*+*^**,255[M+H-NH_2_]**^*+*^**,237[M+H-NH_2_-CO]**^*+*^**	C_16_H_17_NO_3_		Benzylisoquinoline alkaloid		*LP*
	2	0.4061	7.50	Lotusine	314.1801		314[M+H]**^*+*^**,269[M+H-NH_2_CH_3_CH_3_]**^*+*^**	C_19_H_24_NO_3_^+^		Benzylisoquinoline alkaloid		*LP*
	3	(NS **^*b*^**) < 0.1	8.60	4’-methylcoclaurine	298.1458		298[M+H]**^*+*^**,283[M+H-NH_2_]**^*+*^**,251[M+H-NH_2_-CH_3_OH]**^*+*^**	C_18_H_21_NO_3_		Benzylisoquinoline alkaloid		*LP*
	6	0.4206	14.07	Isoliensinine **^*a*^**	611.3080		568[M+H-CH_3_-CO]**^*+*^**,297[M+H-C_18_H_20_NO_4_ **^*]*^** ^+^,192[M+C_26_H_28_NO_4_]**^*+*^**	C_37_H_42_N_2_O_6_		Bisbenzylisoquinoline alkaloid		*LP*
	7	0.2860	15.14	Liensinine	611.3107		568[M+H-CH_3_-CO]**^*+*^** ,283[M+H-C_19_H_22_NO_4_]**^*+*^**,206[M-C_25_H_26_NO_4_]**^*+*^**	C_37_H_42_N_2_O_6_		Bisbenzylisoquinoline alkaloid		*LP*
	8	0.2421	17.44	Neferine **^*a*^**	625.3271		582[M+H-CH_3_-CO]**^*+*^**,313[M+H-C_19_H_22_NO_3_]**^*+*^**,297[M+H-C_19_H_22_NO_4_]**^*+*^**,206[M-C_26_H_28_NO_4_]**^*+*^**	C_38_H_44_N_2_O_6_		Bisbenzylisoquinoline alkaloid		*LP*
	9	0.4742	19.32	Nuciferine **^*a*^**	296.1649		265[M-NH_2_CH]**^*+*^**,250[M-NH_2_CH_3_-CH_3_]**^*+*^**,234[M-NH_2_CH_3_-CH_3_OH]**^*+*^**	C_19_H_21_NO_2_		Aporphine alkaloid		*LL*
	10	0.5091	22.90	β-sitosterol	415.6931		415[M+H]+,396[M^+^-H_2_O]**^*+*^**,255[M^+^-H_2_O-C_10_H_21_]**^*+*^**,213[M^+^-H_2_O-C_16_H_23_]**^*+*^**	C_29_H_50_O		Steroid		*LR*
	12	0.3395	19.17	roemerine	280.1298		249[M-NH_2_CH_3_]**^*+*^**,219[M-NH2CH_3_-CH_2_O]**^*+*^**	C_18_H_17_NO_2_		Aporphine alkaloid		*LL*
	13	0.2110	12.70	4’-Methyl-N-methylcoclaurine (MMC)	314.1735		299[M+H-CH_3_]**^*+*^**,283[M-NH_2_CH_3_]**^*+*^**,237[M-NH_2_CH_3_-CH_3_]**^*+*^**	C_19_H_23_NO_3_		Benzylisoquinoline alkaloid		*LP*
	14	0.2320	13.21	Armepavine	314.1730		283[M-NH_2_CH_3_]**^*+*^**,252[M-NH_2_CH_3_-CH_3_OH]**^*+*^**	C_19_H_23_NO_3_		Benzylisoquinoline alkaloid		*LP*
	16	0.2474	15.07	O-nornuciferine	282.1489		251[M-NH_2_CH_3_]**^*+*^**,236[M-NH2CH_3_-CH_3_]**^*+*^**,208[M-H_2_NH-CH_3_-CH_3_-CO]**^*+*^**	C_18_H_19_NO_2_		Aporphine alkaloid		*LL*
**B**	**Peak NO.**	**Significance**	**t_R_(min)**	**Identification**		**MS [M-H]^-^**	**HPLC-ESI-*MSn* m/z**		**Composition**	**Substance Class**	**MAX.**	
	4	0.3195	10.87	Apigenin-6-C-α-L-glucopyanosyl-8-C-β-D-glucopyranoside(AGG)		593.1747	503[M-H-C_3_H_6_O_3_]^-^,473[M-H-C_4_H_8_O_4_]^-^,443[M-H-C_5_H_10_O_5_]^-^,383[M-H-C_4_H_8_O_4_-C_4_H_8_O_4_]^-^,353[M-H-C_4_H_8_O_4_-C_5_H_10_O_5_]^-^,268[M-H-C_6_H_11_O_5_-C_6_H_11_O_5_]^-^		C_27_H_30_O_15_	Flavone C-glycoside	*LP*	
	5	0.3981	13.18	Apigenin-6-C-α-L-arabofuranosyl-8-C-β-D-glucopyranoside(AAG)		563.1326	563[M-H]^-^,443[M-H-C_4_H_8_O_4_]^-^,413[M-H-C_5_H_10_O_5_]^-^,268[M-C_6_H_11_O_5_-C_5_H_9_O_4_]^-^		C_26_H_28_O_14_	Flavone C-glycoside	*LP*	
	11	0.2701	15.28	Luteoloside		447.0984	447[M-H]^-^,285[M-H-C_6_H_11_O_5_]^-^		C_21_H_20_O_11_	Flavone glycoside	*LL*	
	15	0.4147	18.56	unknown		727.1881	607,591		/	/	*LP*	
	17	0.2907	14.75	Hirsutrin **^*a*^**		463.0750	927[2M-H]^-^,301[M-H-C_6_H_10_O_5_]^-^		C_21_H_20_O_12_	Flavone O-glycoside	*LL*	
	18	0.2360	14.30	Hyperin		463.0771	927[2M-H]^-^,301[M-H-C_6_H_10_O_5_]^-^		C_21_H_20_O_12_	Flavone O-glycoside	*LL*	

***^a^*** *Verification of standard products* ***^b^*** *NS*: no significance

Based on the Q-TOF/MS information, the majority of the difference ingredients were mainly alkaloids and flavonoid glycosides, and the relative retention time (R_t_), UV spectra and ultraviolet absorption bands are presented in Table 1(A: in positive-mode, B: in negative-mode). The alkaloids of the difference ingredients were deleted in the positive mode (Table 1A), while flavone mainly showed response values in the negative mode (Table 1B). The peak positions of Compounds **1**, **2**, **3**, **6**, **7**, **8**, **9**, **12**, **13**, **14** and **16**, in wavelength absorption, were approximately 193 (Max.), 227, and 282 nm, and this result was consistent with previous literature [27]. According to the mass spectra and the MS/MS fragments in the positive-ion mode, these compounds were identified to be isoquinoline alkaloids. One of the ion sets was the [M+1]^+^ ion m/z 625, and the molecular weight of this substance was presumed to be 624. The fragments 313[M+1-C_19_H_22_NO_3_]^+^ and 297[M+1-C_19_H_22_NO_4_]^+^ were observed for this substance, and this component was identified as Neferine [28]. Compounds **4** and **5** were identified as flavone C-glycosides. The UV absorption wavelengths were 228, 271, and 338 nm [29], and the fragments of component **4** ([M-1-120]^-^, [M-1-90]^-^ and [M-1-30]^-^) were typical ion fragments of a flavone C-glycoside [30]. This component was identified as Apigenin-6-C-α-L-glucopyanosyl-8- C-β-D-glucopyranoside (AGG). Compounds **11**, **17**, **18** also belonged to the group of flavone O-glycosides. Compound **10** was β-sitosterol and had no ultraviolet absorption. According to the fragments and cleavage rule, this compound was speculated to be the only steroid. These data are presented in Table 1(A).

### 2. Evaluation of the bioactivity of the NF-κB inhibitors

**Figure 2:**
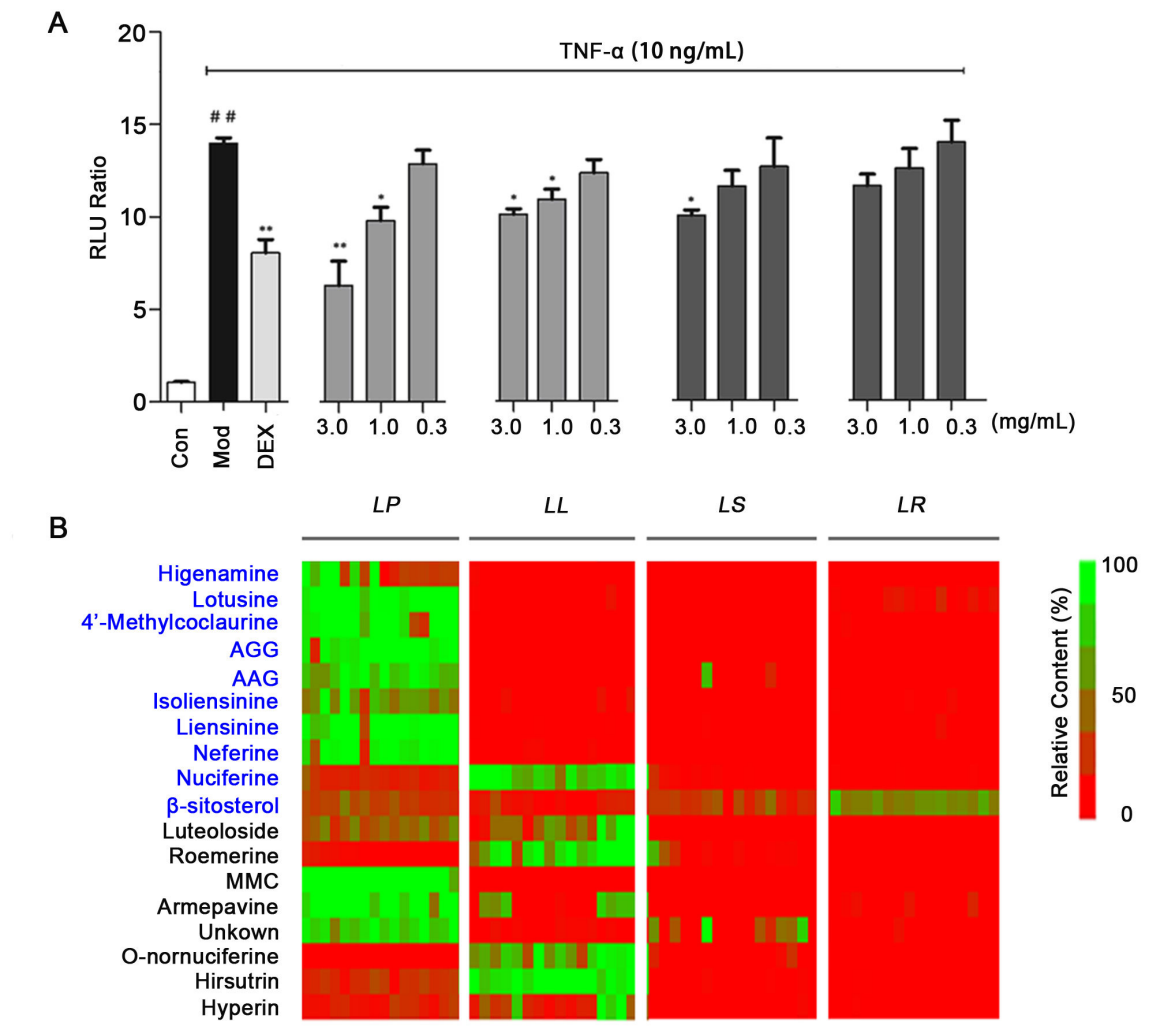
Effects of different organs of *LN* on NF-κB inhibition (A) and the heat map (B). **A**: The levels of NF-κB in TNF-α stimulated HEK 293 cells. Values are presented as the mean ± SEM, *n* = 5 for each group. **p <0.05 *vs* the group that was treated with TNF-α in the absence of extracts and drugs, ^##^ *p* < 0.05 *vs* the Control group; **B**: The relative content of each marker ingredient in different organs. Zero content was expressed as red and largest content (100%) as green.

The effects of the extracts from the different organs of *LN* on NF-κB were first evaluated at the cellular level using a dual-luciferase reporter assay system. As shown in Figure 2(A), the positive Dex (10^-5^ mol/L) significantly inhibited TNF-α-induced, NF-κB production (p<0.01). The results show that the high and middle dosage groups of *LP* significantly inhibited NF-κB, but the high dosage groups of *LL* and *LS* showed little effect on anti-inflammation. This finding demonstrated that the *LP* was an anti-inflammatory agent at the cellular level and was consistent with its use in TCM. 

**Figure 3:**
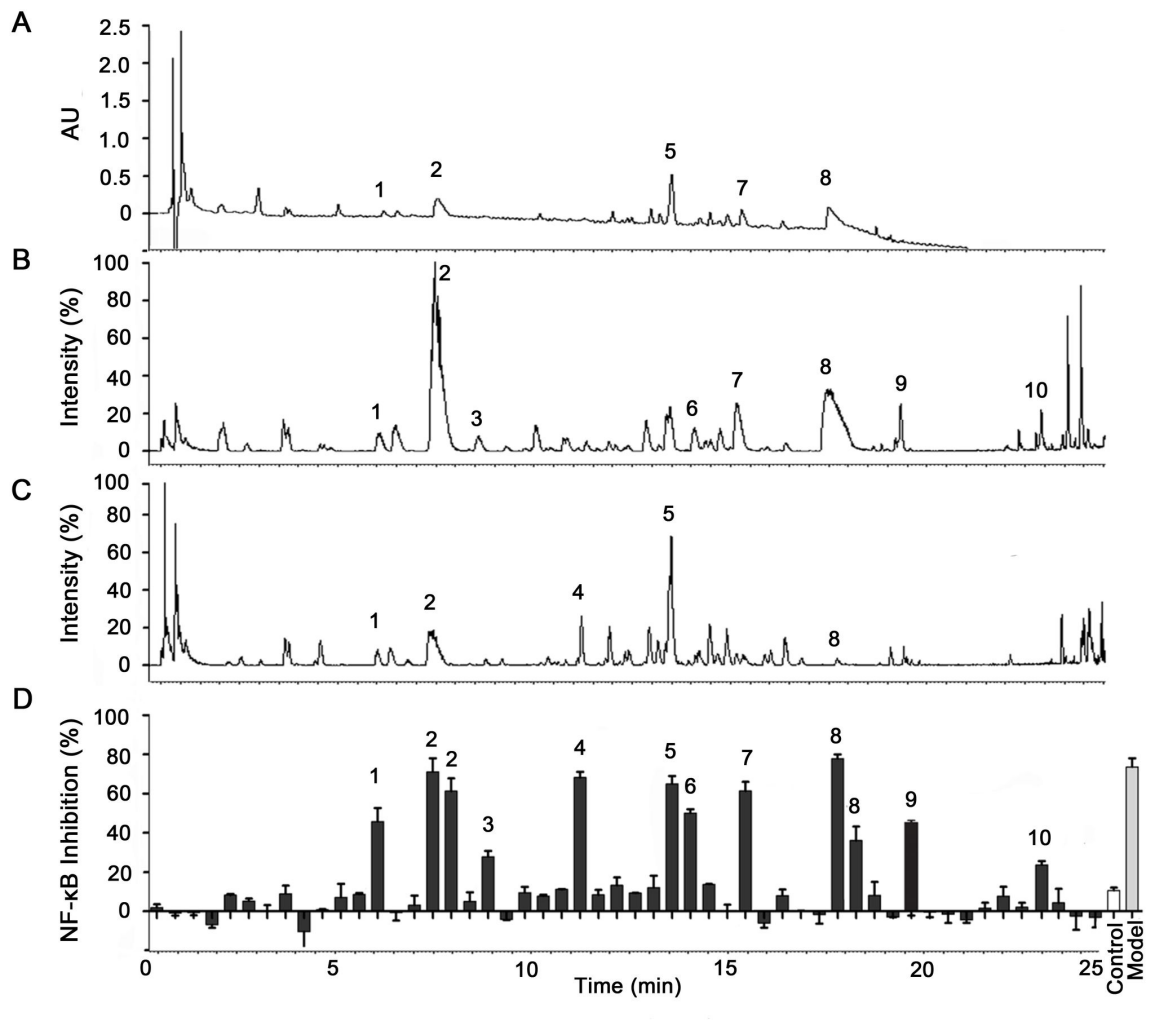
UPLC-Q-TOF/MS and bioactivity analyses of *LP*. (**A**) UPLC-UV chromatograms of LP TIC data in (**B**) the positive and (**C**) negative ESI modes. Bioactivity chromatograms were obtained via the dual-luciferase reporter assay system for (D) NF-κB inhibition. NF-κB inhibition values are presented as the mean ± SEM, *n* = 5 for each group. The peak numbers are consistent with those in Table 1(A and B).

**Figure 4:**
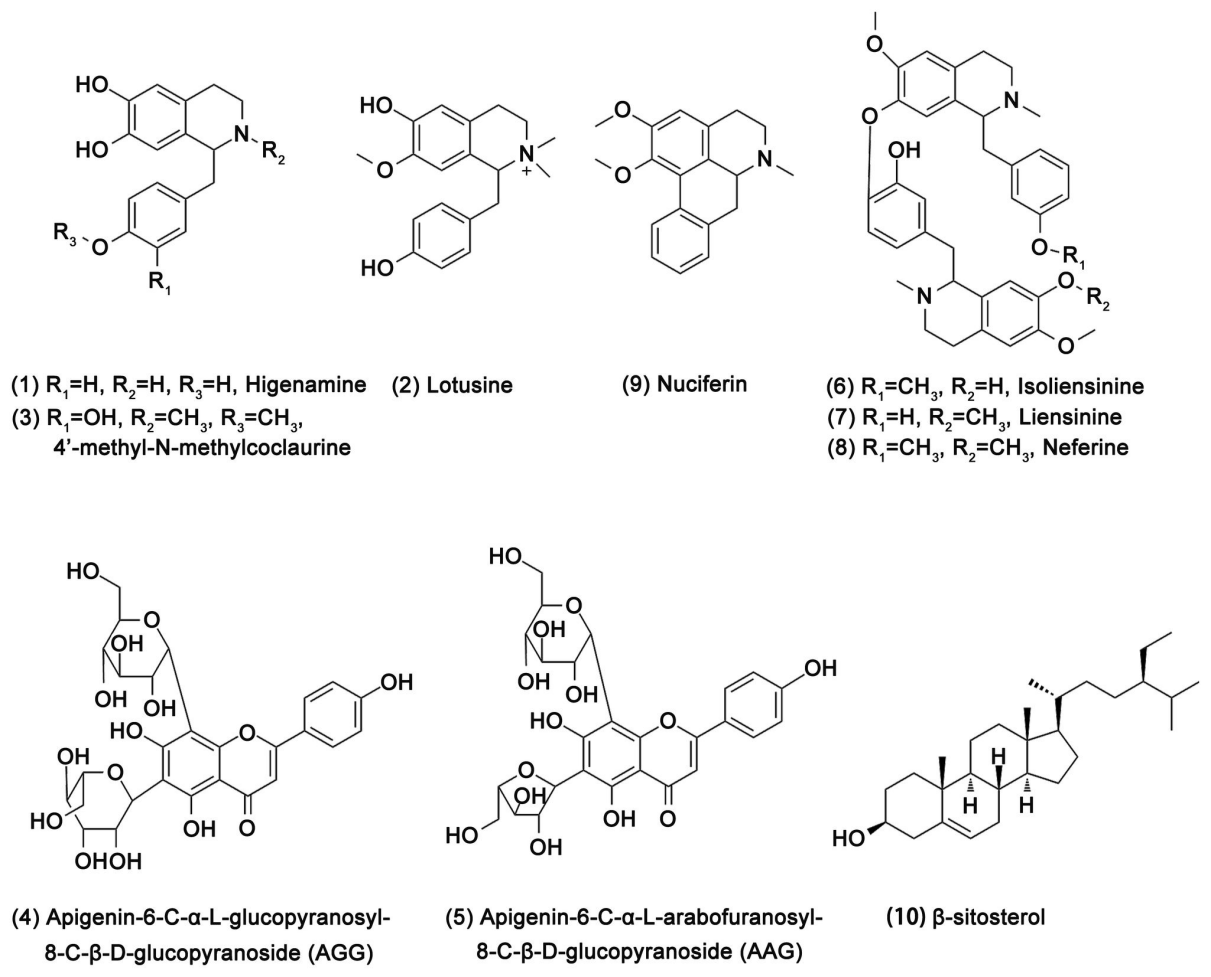
Chemical structures of the bioactive compounds in the *LP.*

As the above result indicated, the *LP* extraction was considered a source of potential NF-κB inhibitors. Therefore, bioactivity-guided fingerprinting was applied to determine the CMB that was responsible for the NF-κB inhibitory effect by *LP*, and *LP* extraction was used for further separation and identification by UPLC-Q/TOF MS analysis. The bioactivity fingerprint for *LP* is shown Figure 3. Nearly all of the NF-κB inhibitors were alkaloids, two compounds were identified as Flavone C-glycosides (compound **4**, **5**), and compound **10** was a steroid. In Table 1, which contains the Q/TOF MS data, these compounds were identified as Higenamine, Lotusine, 4’-methylcoclaurine, Apigenin-6-C-α-L-glucopyanosyl-8-C-β-D-glucopyranoside (AGG), Apigenin-6-C-α-L-arabofuranosyl-8-C-β-D-glucopyranoside (AAG), Isoliensinine, Liensinine, Neferine, Nuciferine and β-sitosterol. The chemical structures of the bioactive compounds are shown in Figure 4.

**Figure 5:**
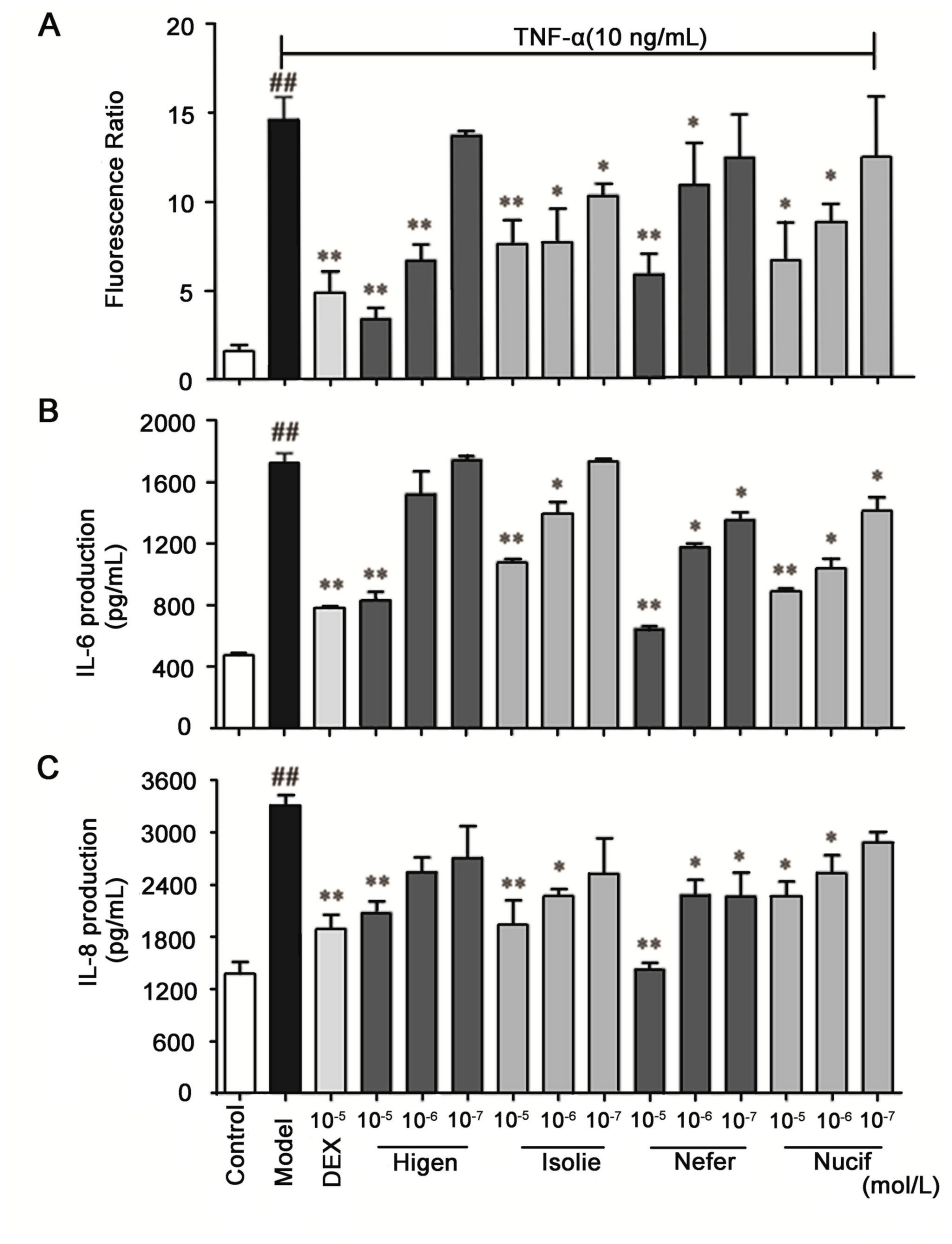
Confirmation of the bioactive compounds from the *LP* in TNF-α-induced BEAS-2B cells. (**A**) Effects of the potential NF-κB inhibitors by the dual-luciferase reporter assay system, effects of the representatives of four NF-κB inhibitor types on (**B**) IL-6 and (**C**) IL-8 expression. Each bar represents the mean ± SEM. *n* = 5, **p < 0.05 *vs* the group that was treated with TNF-α in the absence of extracts and drugs, and ^##^ *p* < 0.05 *vs* the Control group. One (*) or two (**) asterisks indicate *p* < 0.05 or *p* < 0.01, respectively.

The confirmation method of BEAS-2A for NF-κB was same as HEK 293 cell. After stimulation by TNF-α, NF-κB protein levels were abundantly expressed in transfected BEAS-2B cells. Simultaneously, this stimulation caused a significant increase in the expression of IL-6 and IL-8 in the cells, and Higenamine, Isoliensinine, Neferine and Nuciferine were able to reduce this overexpression (*P*<0.05) (Figure 5). This result demonstrated that these four compounds inhibited NF-κB. The dual-luciferase reporter assay system can be successfully used to screen NF-κB inhibitors from plant extracts, and previous studies have indicated that Neferine and Higenamine could block NF-κB activity and showed a significant anti-inﬂammatory effect [31,32]. While, Isoliensinine and Nuciferine were considered new NF-κB inhibitors for the first time, few reports have mentioned their anti-inflammation properties. According to the structure of these NF-κB inhibitors, we inferred that the benzylisoquinoline-, bisbenzylisoquinoline- and aporphine-type alkaloids might generally possess anti-inﬂammatory effects. Due to the significant effect that this finding has on the anti-inflammatory field, more attention should be paid to these compounds.

### 3. Comprehensive evaluation and analysis

In TCM, different portions of the same plant often have different purposes. Although the same or similar types of chemicals were found in these organs of the plant, the biological activity of each part could be emphasized in a particular part [33]. Determining the type and content of the active compounds was critical because doing so could identify the type of activity on a pharmaceutically acceptable basis. Therefore, it was of great significance to identify the difference ingredients for herbal medicinal applications and to separate the active ingredients [34]. 

**Figure 6:**
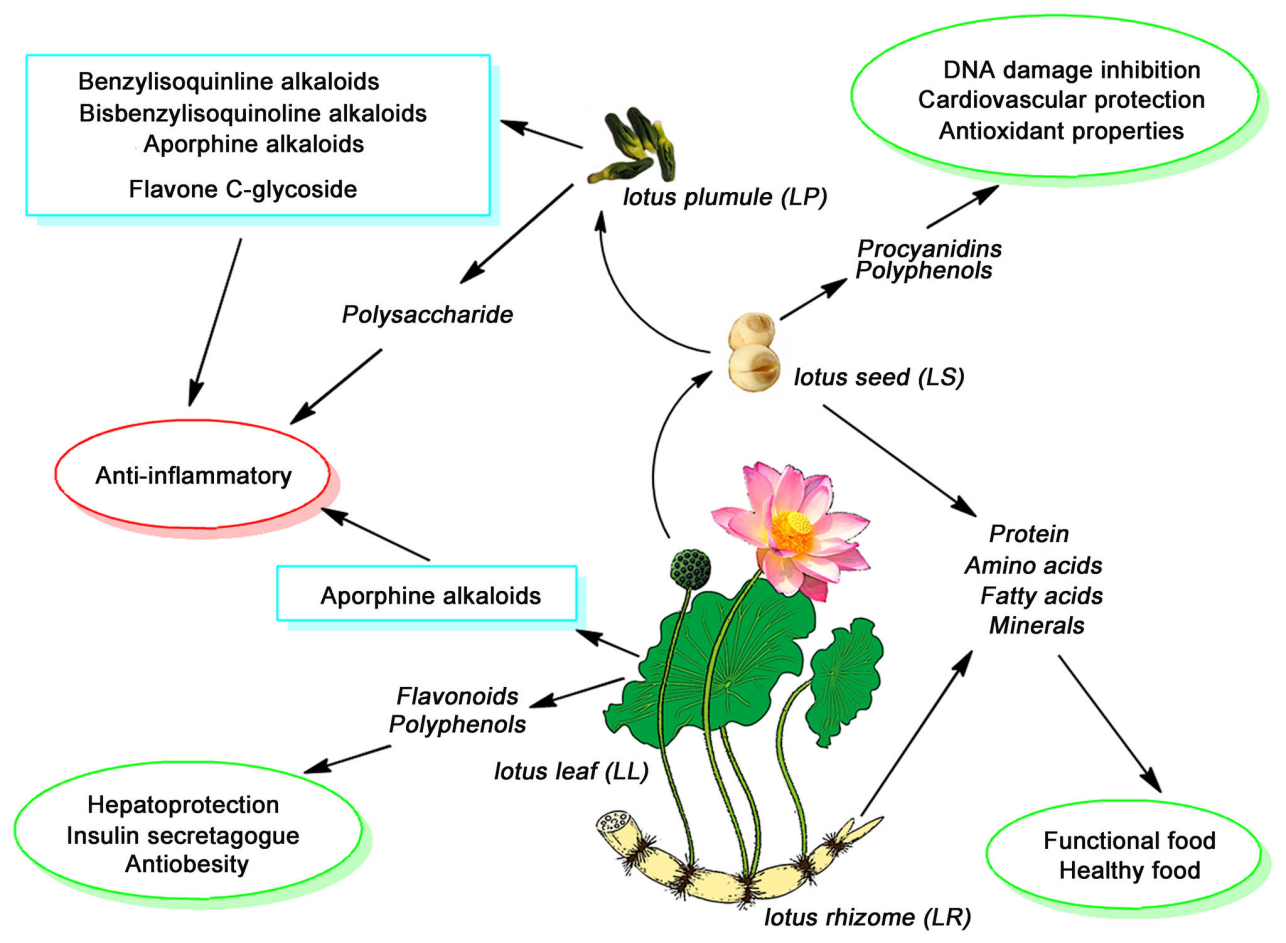
The organ-active-ingredients application for *LN* organs.

We identified approximately 17 main difference ingredients in the *lotus*, and each of these ingredients belonged to difference chemical types and was rich in different organs. The relative content of each compound in different organs of the plant was visualized in heat map as shown in Figure 2 (B). In Figure 2 (B), the target compounds (totally 18 compounds) were the difference ingredients obtained from PCA and the bioactive compounds screened by dual-luciferase reporter assay system. The color code indicated “zero” (red) and “the largest” (green) content of each compound based on the normalized data. The k-means value was set at 4 and the data was classified into four clusters (i.e., *LP, LS, LL* or *LR*). According to the heat map, the first ten compounds (NF-κB inhibitors screened by dual-luciferase reporter assay system) were marked with a blue font. Eight NF-κB inhibitors were most abundant in *LP*, which was responsible for the best anti-inflammatory activity of *LP*. Other target ingredients distributed in 4 different organs, relating to the various bioactivities of these organs. Importantly, these compounds also had various biological activities that resulted in each organ having its respective medicinal value. The comprehensive evaluation and analysis of the organ-active-ingredient application for *LN* organs is displayed in Figure 6.

Figure 6 summarizes the applications of different organs that were found in the literature, and this study, which was performed using several research methods, integrated a large number of samples. According to the literatures, *LP* has been used to treat inflammation-related diseases, but other parts of the same plant have not been used in this manner. The results in this study showed that the bioactivity of *LN* was closely related to the type and content of the difference ingredients from the *LN, LP, LS*, and *LL* (Table 1). Based on the dual-luciferase reporter assay system, nine of the ten NF-κB inhibitors from *LN* belonged to the key difference ingredients that were found in different parts of the plant, but only compound **3** (4’-methylcoclaurine) was an exception because no significant difference emerged in the Loading plots. Nearly all of the NF-κB inhibitors belonged to the benzylisoquinoline, bisbenzylisoquinoline and aporphine alkaloid groups, two compounds were Flavone C-glycosides (compound **4**, **5**), and compound **10** was a steroid. Based on the base-peak intensity (BPI) chromatograms and the heat map, it was also clear that eight of these active ingredients were most abundant in the *LP*, which fully explained why *LP* exhibited the best anti-inflammatory activity. In a previous study, polysaccharide from *LP* was evaluated for its anti-inflammatory effects [35,36], which suggested that the differences between the anti-inflammatory effects of the four organs were mainly caused by the principal components and polysaccharides.

According to the PCA and heat map results, several difference ingredients were most abundant in the *LL*, such as the compounds that were found at the **9, 12**, and **16** peaks, which corresponded to the aporphine alkaloids. Therefore, *LL* also showed some anti-inflammatory effects (Figure 2). However, this organ mainly contains flavonoids and phenolic compounds (compound **11**, **17**, **18**), and it was reported that it held an obvious hepatoprotective, insulin secretagogue and antiobesity effect [33,37,38]. The leaf of this plant has also been used as diet tea in China. 

While the medicinal effects of the other two organs (*LS* and *LR*) was not dominant. When compared to *LP* and *LR*, these organs possessed a few active constituents (Compound **10**) that have been used as edible agricultural products [39]. This finding coincided with the PCA results. The two organs mainly contain nutrients, such as proteins, amino acids, unsaturated fatty acids and minerals, which are mainly used as functional food and healthy food [10,11]. However, oligomeric procyanidins and phenolic compounds were found in the *L*S. Therefore, *LS* may possess DNA damage inhibition, cardiovascular protection and antioxidant properties [40,41]. 

## Conclusion

A large amount of data was obtained from different organs of *LN* and was processed simultaneously by integrated instrument analysis, multivariate statistical analysis and biological research tools. Samples were accurately discriminated, and **17** difference marker compounds were identified by UPLC/Q-TOF-MS with PCA. Due to its difference chemical basis, the anti-inflammatory activity of the *LP* was superior to those of other parts of the plant. Finally, several ingredients with NF-κB inhibitory activity were identified using biochemical joint technology. PCA results indicated that most of these compounds were isoquinoline alkaloids or phenol flavonoids (glycosides) and were most abundant in *LP*, as same as indicated by the heat map results. This finding suggests that *LP* is more plausible for use in anti-inflammation. Cumulatively, these results demonstrated that the tandem technical application showed significant value in identifying the CMB of different plant samples. This result lays a good foundation for further research on the chemical structure characteristics and mechanism of anti-inflammation in the other plants. Likewise, the research strategy in this study is simple and efficient and could be applied in plant physiology and biochemistry.
